# A closer look at regional differences in euthanasia practices in the Netherlands

**DOI:** 10.1007/s43999-025-00069-x

**Published:** 2025-07-23

**Authors:** Wieke M. R. Ligtenberg, Theo A. Boer, A. Stef Groenewoud

**Affiliations:** 1 Theological University Utrecht, Utrecht, The Netherlands; 2 Protestant Theological University, Utrecht, The Netherlands; 3 IQ Health, Radboudumc, Nijmegen, The Netherlands

**Keywords:** End of life care, Euthanasia and assisted suicide, Medical practice variation

## Abstract

**Background:**

In research on practice variation, the body of knowledge on regional differences in the incidence of euthanasia is limited, and important questions have remained unanswered until now.

**Objective:**

This paper aims to gain insight in the differences between euthanasia practices in high-incidence regions and low-incidence regions, by looking at (potential differences in) a) patient characteristics; b) timelines and the process of euthanasia; c) the setting in which euthanasia takes place; and d) morally relevant themes.

**Methods:**

This explorative study uses a unique and fully anonymized dataset based on notes made by one of the authors (TAB) during a period of nine years in which he was an ethicist in a Dutch Euthanasia Review Committee. We analyzed these data using descriptive statistics and testing for statistical significance of differences in euthanasia practices in high-incidence regions and low-incidence regions.

**Results:**

Some significant differences were found between high and low-incidence regions. Compared to low-incidence regions, high-incidence regions were characterized by patients being older at time of death, a shorter time span between patients’ first euthanasia request and their eventual death, patients more often having a GP as a consulting doctor, and euthanasia more frequently being the main dying means (as opposed to assisted suicide). The low incidence regions had somewhat younger patients, more patients with dementia, a longer life expectancy, more psychiatrists as consulting doctors, and more assisted suicides compared to the higher incidence regions.

**Conclusion:**

This study adds new insights to the existing literature on (regional differences in) end-of-life care, with a specific focus on euthanasia. Until now, euthanasia practices have mainly been studied at national levels. National data show significant differences between regions. The black box of local practices has not been opened before. Our results have implications for practice, as they may inform discussions on appropriate care at the end of life in general, and euthanasia in particular.

## Introduction

Worldwide there are 26 jurisdictions with some form of legalized assisted dying (euthanasia and/or assisted suicide). They include six states of Australia, the countries of Belgium, Canada, Colombia, Ecuador, Luxemburg, the Netherlands, New Zealand, Spain, Switzerland, and 10 states (plus the District of Columbia) in the United States [18, 26]. Following a makeshift law of 1994, the Netherlands was the first country in the world to legalize euthanasia in 2002 (Table 1: legal requirements). In the Dutch context both euthanasia and physician assisted suicide are allowed. Euthanasia is the act in which a physician administers the lethal drugs to end a patient’s life. In physician assisted suicide the patient self-administers the lethal medication that was prescribed by a physician [20]. In almost all cases the lethal drugs are administered by the physician through an injection (euthanasia, 98%), only in a few cases the patient takes the lethal dose themselves, through a drink (physician assisted suicide, 2%) [23].

Since 2002 the Dutch euthanasia practice is rapidly evolving. There are rising numbers [17, 24], a broadening of pathologies underlying a euthanasia request [15, 17, 21, 29], and profound regional differences [10, 17, 19].

**Table 1:** Legal requirements [28]

Substantive requirements:
- A patient request that is voluntary and well-considered
- The patient’s suffering must be unbearable and hopeless
- The physician must inform the patient about their health condition and prospects
- The physician and patient must come to the belief that there is no reasonable prospect of improvement in the patient’s situation
- The physician must terminate the life in a medically appropriate way
Procedural requirements:
- The attending physician must consult another physician before proceeding
- Afterwards, the physician must submit a euthanasia case for review to one of five regional Euthanasia Review Committees

Despite these seemingly clear guidelines, it still appears that euthanasia practice shows considerable variation between Dutch regions [10, 19]. However, our knowledge of regional differences in euthanasia, and what lies behind them, is limited. Although there is a body of research on differences in end-of-life care practices in the fields of sociology and anthropology, these insights are often at the level of cultures and societal groups. Practice variation research investigates differences in health care practices. Most studies on practice variation focus on identifying and mapping variations in end-of-life care (decisions) in general, only some specifically on assisted dying.

Previous studies on regional variation in end-of-life care in general from the United States, Belgium, England, Israel and Switzerland, found variations in patient characteristics, end of life interventions and place of death [27]. Studied patient characteristics included age, living arrangements, comorbidity [27], ethnicity, and cause of death [3]. Variations in interventions during the last phase of life were shown by looking at (emergency) hospital admission, specialist visits, palliative care needs [27], the use of specific medical services such as feeding tubes [7–9] and end of life decisions such as intensified alleviation of pain, withdrawing life prolonging treatment, continuous deep sedation and physician assisted dying [3, 6, 14]. For place of death, variations were found in the proportion of people who died at home, in a hospital, in a hospice or in a nursing home [3]

Cohen et al. [3] found that the incidence of most end-of-life decisions (such as intensified alleviation of pain, withholding or withdrawing treatment and physician assisted death) was lower in metropolitan Brussels compared to non-metropolitan Flanders, except for the use of life-ending without explicit patient request, which was higher in Brussels [3]. Ganz et al. [6] found significant associations between end-of-life decisions and regions (in norther/central/southern Europe and Israel) in the context of intensive care. Hurst and her Swiss colleagues (2018) compared the prevalence of medical end of life decisions (MELDs) between German, French, and Italian speaking regions. They found a greater reluctance to forgo life-prolonging treatment in French and Italian speaking regions, suggesting this might have to do with cultural differences reflected in a more family oriented approach (Italian speaking region) and a more technology-oriented approach (French speaking region) [14].

Different authors suggest that the found differences might occur because of characteristics of the population, differences in patient-caregiver relationships, different cultural attitudes towards death or differences in access to medical services [3, 6–9].

Within the field of practice variation research, very little work has been done on practice variation in assisted dying specifically. Chambaere and colleagues (2010) describe differences between 552 French and 149 Dutch speaking physicians in Brussels. Euthanasia was more often performed by Dutch- than French-speaking physicians, however this difference was not statistically significant. Continuous deep sedation was performed significantly more often by French speaking physicians. Since their research was done in the bilingual city of Brussels, the found differences were irrespective of geographical location but rather had to do with different language communities [2]. On the national level however, geographical variation was found between Flanders and Wallonia, with a tendency towards euthanasia in Flanders and more continuous deep sedation in [30]. Cohen and colleagues (2012) delved into these cultural differences to explain differences in attitudes and practices between Flanders and Wallonia. It appeared that although the acceptance of the practice of euthanasia, both by doctors and the general public, did not differ between the regions, there were large differences in the proportion of physicians that had received requests and the willingness/reluctance to perform euthanasia (overall more requests and willingness in Flanders than in Wallonia) [4]. Groenewoud et al. [10] found geographical variation in incidence of euthanasia in the Netherlands. At the level of municipalities, differences were found with factor scores between 27 and 17. Factors that were associated with regional variation were age, church attendance, political preferences, availability of voluntary workers, income and self-experienced health [10]. Thus far practice variation research has investigated variables that have to do with health disparities, and preference differences at the municipal level. No previous study has looked at actual cases from euthanasia practice at the municipal level.

Previous research has primarily focused on *identifying* and *mapping* regional differences. More precise characteristics of the different practices often remain unclear. In our research, like Cohen and colleagues (2012), we take a closer look at euthanasia practices in specific regions. We are opening the black box of local euthanasia practice. In this study we will use a unique dataset to look at – and compare – characteristics of euthanasia cases in low and high-incidence regions in the Netherlands. By analyzing data from different Dutch municipalities, we answer our research question: *What characterizes the patients, the process and the setting of euthanasia in high/low incidence regions?* To what degree do a) patient characteristics; b) the timelines and the process of euthanasia; c) the setting in which euthanasia takes place; and d) morally relevant themes differ between regions? In this study we aim to shed light on aspects of local euthanasia practices that have so far remained largely out of sight.

## Methods

This is an explorative mixed method study with longitudinal data on euthanasia cases (2008–2014), encompassing both quantitative data on patient characteristics, timelines, and information about the setting in which euthanasia took place, as well as qualitative data on morally relevant themes.

### Data collection and variables

According to Dutch law, a doctor should report each case in which euthanasia has taken place to a Regional Review Committee (RRC). Committees consist of a lawyer, an ethicist and a doctor. Together they evaluate whether the physician handled the case with due care [16].

In this study we used a dataset, based on 3700 euthanasia cases, which was created by one of the authors (TAB) during his 9-year membership of the RRC committee region Midden between 2005 and 2014. During this time, he kept personal records of several characteristics of the euthanasia cases. He tracked them in a separate document which led to a database. Amongst other things, the dataset contains quantitative data about the zip code and specialty of the involved physician, several patient characteristics (gender, age, diagnosis, cause of suffering, estimated life expectancy), and information of the euthanasia process (place of death, advance directive, timelines between diagnosis, euthanasia wish and actual life termination).

We define the following variables a bit more precise because they are central to the comparative analysis; earliest euthanasia intention, actual euthanasia request, diagnosis and life expectancy. We define ‘earliest euthanasia intention’ as the first time a patient ever started talking about the wish for euthanasia, either with family, or with their doctor. This information could be found in the extended file of the euthanasia case one of the authors had access to (TAB). The ‘actual euthanasia request’ is the formal request by a patient with their doctor. For the variable ‘diagnosis’ we followed the categories that are used by the RCC’s: cancer, age-related, heart-, lung-, neurological, and psychiatric diseases [23]. ‘Other’ was used when more than one or two diagnoses were found, or when the diagnoses did not fit on of the diagnoses categories. Lastly, the variable life expectancy, which is about the natural life expectancy as denoted in the files (since after 2011 a question about this was left out from the formal questionnaire, the estimation after 2011 is based upon an interpretation by TAB on the basis of information from the medical records).

Besides quantitative data, the dataset contains morally relevant notes. These notes are directly or indirectly linked to the legal criteria in Dutch law generally considered the morally most essential ones. Although the due care criteria have remained the same since 1994, developments in the past two decades have led to extra attention for how these criteria work out in practice [12, 24]. The criterion *‘a voluntary and informed request by a competent patient’* justifies special attention for indications of pressure from relatives or caregivers, for references to the specific interpretation and weight of the patient’s autonomy, and for references to the euthanasia process being transferred to a different doctor than the attending physician; the *‘unbearable suffering’* criterion justifies special attention for indications of existential and social suffering (loneliness); and the *‘no prospect of improvement’* criterion justifies attention for indications that viable treatment- or palliative options were rejected. We included these observations, which were included in the notes because of their potential importance for the committee’s ruling, as qualitative data. For the purpose of this study, we only looked for such morally relevant comments insofar as their occurrence in the different regions varied meaningfully at a first glance, as this may have potential relevance for understanding regional differences.

The data were processed anonymously, and the dataset contains no personally identifiable information (neither in terms of the patient nor of the euthanizing doctors).

### Sample

Region ‘Midden’ is one of the five euthanasia regions in the Netherlands, and includes very different communities: urban and secularized areas (such as Almere, Utrecht, and the Arnhem-Nijmegen area); mixed, semi- and suburban areas; and rural and more traditional areas (including some places known as part of the ‘Bible belt’). As such they offer a sufficient representation of the national variation. The dataset contains data of about 40% of all the euthanasia cases in this region, between 2008 and 2014, based on a random assignment of cases by RTE-secretaries due to rotating membership.

Based on previous research, we know the percentage of euthanasia cases (as a percentage of all deaths) for all Dutch municipalities from 2013 to 2017 [10]. To avoid small sample variation, we selected the municipalities in the region ‘Midden’ with more than 500 deaths (with or without euthanasia) in 5 years (*n* = 101). From these, two quintiles were selected: the top 20 municipalities with the highest euthanasia rate, and the bottom 20 municipalities with the lowest euthanasia rate. We only included records in the dataset that contained the variables diagnosis and natural life expectancy (this was consistently kept from 2008, hence records from that year onwards were included).

Altogether, this resulted in a selection of 20 high-incidence (4.3–7.5%) and 18 low-incidence (0.5%−1.8%) municipalities (Appendix 1). In total, these municipalities accounted for 830 euthanasia reports from high-incidence municipalities and 153 euthanasia reports from low-incidence municipalities.

### Analyses

#### Quantitative analysis

We performed descriptive statistics (average, mean in absolute numbers and percentages), for all variables for both groups (cases from high-incidence regions (*N* = 830) and from low-incidence regions (N-153)). We first looked at our data, guided by descriptive statistics: MINIMUM MAXIMUM; MEAN; MEDIAN; SKEWNESS, and KURTOSIS, as well as by looking at histograms. We did not follow up with e.g. Shapiro–Wilk testing, because we assumed that the difference between t-testing and e.g. Mann–Whitney tests would not be that significant, given the large number of observations (*N* = 153; 830).

We tested for statistically significance of differences between the high and low-incidence regions. For variables with numeric, continuous data, with a normal distribution, we applied unpaired t-tests. Tables 2, 3 and 4 show for each variable what test was chosen. All analyses were performed using IBM SPSS Statistics 29.

**Table 2:** Patient characteristics

	**Cases from high-incidence regions (*N* = 830)**				**Cases from low-incidence regions (*N* = 153)**						
	N	%	mean	SD	N	%	mean	SD			
**Gender**									χ2	df	Asymptotic Significance (2-sided)
Male	412	49,64%			83	54,25%			1,07	1	0,301
Female	417	50,24%			70	45,75%					
*Missing*	1	0,12%			0	0,00%					
**Age**									t	df	Sig. (2-tailed)
Continuous:	826	99,52%	**70,24**	13,14	153	100,00%	**67,88**	12,76	2,050	977	**0,041**
*Missing*	4	0,48%			0	0,00%					
**Co-morbidities**									χ2	df	Asymptotic Significance (2-sided)
**Dementia**	21	**2,53%**			9	**5,88%**			4,907	1	**0,027**
Several elderly-related problems	28	3,37%			2	1,31%			1,864	1	0,172
Heart disease	58	6,99%			10	6,54%			0,041	1	0,840
Cancer	672	80,96%			124	81,05%			0,001	1	0,981
Lung disease	55	6,63%			5	3,27%			2,543	1	0,111
Neurological problems	48	5,78%			8	5,23%			0,074	1	0,786
Psychiatric problems	19	2,29%			4	2,61%			0,06	1	0,807
**Other**	65	**7,83%**			5	**3,27%**			4,067	1	**0,044**
**# (co)morbidities**									t	df	Sig. (2-tailed)
Continuous:	830	100,00%	1,16	0,45	153	100,00%	1,09	0,33	1,897	981	0,058
*Missing*	0	0,00%			0	0,00%					
**Cause of suffering**									χ2	df	Asymptotic Significance (2-sided)
Existential	316	38,07%			60	39,22%			0,072	1	0,789
Physical	789	95,06%			142	92,81%			1,305	1	0,253
Psychological	26	3,13%			7	4,58%			0,829	1	0,363
Social	40	4,82%			12	7,84%			2,358	1	0,125
Other	0	0,00%			0	0,00%			constant	constant	constant
*Missing*	0	0,00%			0	0,00%

**Table 3:** Process

	**Cases from high-incidence regions (*N* = 830)**				**Cases from low-incidence regions (*N* = 153)**						
**Life expectancy at time of euthanasia (days)**									t	df	Sig. (2-tailed)
**Continuous:**	830	1238,81%	**116,80**	500,47	153	7,35%	**272,57**	1011,70	−2,918	981	**0,004**
*Missing*	0	0,00%			0	0,00%					
**Categories:**									χ2	df	Asymptotic Significance (2-sided)
0–30	676	81,45%			123	80,39%			0,094	1	0,759
31–182	94	11,33%			10	6,54%			3,132	1	0,077
183–730	33	3,98%			7	4,58%			0,119	1	0,730
≥ 731	27	**3,25%**			13	**8,50%**			9,1	1	**0,003**
*Missing*	0	0,00%			0	0,00%					
**Time diagnosis – euthanasia (days)**									t	df	Sig. (2-tailed)
**Continuous:**	692	83,37%			118	77,12%			−0,304	808	0,761
*Missing*	138	16,63%			35	22,88%					
**Categories:**									χ2	df	Asymptotic Significance (2-sided)
0–30	12	1,45%			2	1,31%			0,018	1	0,894
31–182	188	22,65%			26	16,99%			2,428	1	0,119
183–730	250	30,12%			43	28,10%			0,251	1	0,616
731–3650	196	23,61%			39	25,49%			0,25	1	0,617
≥ 3651	46	5,54%			8	5,23%			0,024	1	0,876
*Missing*	138	16,63%			35	22,88%					
**Time earliest intention – euthanasia (days)**									t	df	Sig. (2-tailed)
**Continuous:**	425	51,20%			67	43,79%			−0,589	490	0,556
*Missing*	405	48,80%			86	56,21%					
**Categories:**											
0–30	80	9,64%			16	10,46%			0,098	1	0,754
31–182	158	**19,04%**			17	**11,11%**			5,545	1	**0,019**
183–730	102	12,29%			21	13,73%			0,243	1	0,622
731–3650	63	7,59%			10	6,54%			0,209	1	0,648
≥ 3651	22	2,65%			3	1,96%			0,248	1	0,618
*Missing*	405	48,80%			86	56,21%					
**Time request – euthanasia (days)**									t	df	Sig. (2-tailed)
**Continuous:**	402	48,43%			61	39,87%			0,448	461	0,654
*Missing*	428	51,57%			102	66,67%					
**Categories:**									χ2	df	Asymptotic Significance (2-sided)
0–1	17	2,05%			2	1,31%			0,374	1	0,541
2–7	146	17,59%			24	15,69%			0,327	1	0,567
8–14	103	12,41%			12	7,84%			2,608	1	0,106
15–30	57	6,87%			7	4,58%			1,115	1	0,291
31–182	66	7,95%			13	8,50%			0,052	1	0,820
≥ 183	13	1,57%			3	1,96%			0,126	1	0,723
*Missing*	428	51,57%			102	66,67%					
**Time first intention – diagnosis (days)**	**Cases from high-incidence regions (*N* = 830)**				**Cases from low-incidence regions (*N* = 153)**				t	df	Sig. (2-tailed)
	422	50,84%	413,78	2155,74	65	42,48%	409,22	3195,73	0,015	485	0,988
*Missing*	408	49,16%			88	57,52%					
**Categories:**									χ2	df	Asymptotic Significance (2-sided)
≤ −3651	17	2,05%			3	1,96%			9,506	9	0,392
−3650 tot −731	24	2,89%			3	1,96%					
−730 tot −183	14	1,69%			2	1,31%					
−182 tot −31	10	1,20%			0	0,00%					
−30 tot 0	33	3,98%			6	3,92%					
0 tot 30	46	5,54%			3	1,96%					
31 tot 182	79	9,52%			8	5,23%					
183–730	88	10,60%			17	11,11%					
731–3650	90	10,84%			21	13,73%					
> 3651	21	2,53%			2	1,31%					
*Missing*	408	49,16%			88	57,52%

**Table 4:** Setting

	**Cases from high-incidence regions (*N* = 830)**		**Cases from low-incidence regions (*N* = 153)**				
**Specialty of euthanizing physician**					χ2	df	Asymptotic Significance (2-sided)
Medical specialist (int/ENT/Surg/Urol/Hemat/MDL)	22	2,65%	2	1,31%	6,449	6	0,375
GP	771	92,89%	145	94,77%			
Hospice	1	0,12%	0	0,00%			
Oncologist	14	1,69%	0	0,00%			
Psychiatrist	1	0,12%	1	0,65%			
Geriatrician	19	2,29%	5	3,27%			
*Unknown*	2	0,24%	0	0,00%			
**Specialty of consulting physician**					χ2	df	Asymptotic Significance (2-sided)
Medical specialist (int/ENT/Surg/Urol/Hemat/MDL)	51	6,14%	10	6,54%	15,868	5	**0,007**
GP	681	**82,05%**	120	**78,43%**			
Hospice	0	0,00%	0	0,00%			
Oncologist	20	2,41%	2	1,31%			
Psychiatrist	23	**2,77%**	14	**9,15%**			
Geriatrician	54	6,51%	7	4,58%			
*Unknown*	1	0,12%	0	0,00%			
**Dying means**					χ2	df	Asymptotic Significance (2-sided)
Euthanasia	493	**59,40%**	74	**48,37%**	10,084	3	**0,018**
Physician assisted suicide (PAS)	22	2,65%	4	2,61%			
First PAS, then euthanasia	6	**0,72%**	4	**2,61%**			
*Missing*	309	37,23%	71	46,41%			
**Place of death**					χ2	df	Asymptotic Significance (2-sided)
Home	344	41,45%	54	35,29%	4,608	4	0,330
Hospice	36	4,34%	4	2,61%			
Nursing home	29	3,49%	4	2,61%			
Hospital	18	2,17%	3	1,96%			
*Unknown*	402	48,43%	88	57,52%			

#### Qualitative analysis

Our analysis of the observed morally relevant themes was inspired by what is called ‘summative content analysis’ [13]. We investigated how often the different themes were highlighted in cases from different regions. Differences in coding and our reasoned assumptions in doing so were discussed in the research team. Then we compared the content of the themes that differed mostly and meaningfully between the regions. This led to indications of variation in morally relevant themes between high-incidence and low-incidence areas. In the discussion we reflect on the possible causes of these differences. The quotes we use are anonymized verbatim excerpts of euthanasia files, which were denoted by one of the researchers (TAB) during his period in the Regional Euthanasia Review Committee (RRC). Most of the quotes are from attending physicians (who perform the euthanasia) or from consulting physicians (who provide second opinions).

## Ethical approval

The Medical Ethics Review Committee of VU University Medical Center affirmed on Dec. 20, 2017 that the ‘Medical Research Involving Human Subjects Act’ does not apply to our study and that an official approval by this committee is not required. The Medical Ethics Review Committee of VU University Medical Center is registered with the US Office for Human Research Protections (OHRP) as IRB00002991. The FWA number assigned to VU University Medical Center is FWA00017598. In a letter of March 21, 2018, the Standing Committee on Research and Ethics of the Faculty of Theology and Religious Studies of VU University approves and recommends this study.

## Results

Our database contained a total number of 983 persons that received euthanasia, 830 from high-incidence regions and 153 from low-incidence regions. In accordance with the research questions the results are presented in three sub-sections: patient characteristics; process characteristics; and the settings.

### The patients

#### Gender and age

Half of the individuals in high-incidence regions and 45% of individuals in low-incidence regions were female, whereas 50% respectively 55% were male, which is a more or less comparable (not statistically significant different) distribution of men and women in both groups. Furthermore, individuals that received euthanasia in high-incidence regions were older at their time of death (mean: 70 years) than individuals from low-incidence regions (mean: 68 years) who received euthanasia. This was a statistically significant difference (*p* = 0,041).

#### Diagnosis

In 21 of the 830 cases (2.5%) in the high-incidence regions the person was diagnosed with dementia, and in the low-incidence regions this was 9 of the 153 patients (5.9%). This poses a statistically significant difference (*p* = 0,027). Furthermore, individuals from high-incidence regions were more often diagnosed with ‘other’ diseases than individuals from low-incidence regions (*p* = 0,044), which means that the pathology in high-incidence regions is more diverse than in low-incidence regions. E.g., more than one or two diagnoses were found, or the diagnoses did not fit the diagnoses used by the RRC: cancer, age-related, heart-, lung-, neurological, and psychiatric diseases. For those diagnoses, no significant differences were found.

#### Suffering

We distinguish four categories of suffering: physical (including pain, apnea, care dependence, visual/hearing impairment), psychological/psychiatric, social (including loneliness and grief), and existential (including despair and loss of autonomy). In most patients, two or more of these categories come together (leading to totals far beyond 100%). The most common form of suffering in both high and low-incidence regions is physical (93–95%), followed by existential (38–39%), social (5–8%) and psychological (3–5%).

### The process

#### Life expectancy

The average estimated natural life expectancy (in days) at the time of euthanasia was higher for individuals from low-incidence regions (mean: 273 days) than for individuals in high-incidence regions (mean: 117 days). This is a statistically significant difference (*p* = 0,004).

Of the patients in high-incidence regions, at time of death, 81.5% had a life expectancy of less than one month, 11.3% had a life expectancy of between 2 and 6 months, 4% had a life expectancy of 7 months to two years, and 3.2% had a life expectancy of more than two years. In comparison, in the low-incidence regions there were 8.5% of the patients in the category of more than two years. These are statistically significant more patients in this category than in the high-incidence regions (3.3%).

#### Time between diagnosis and death

The time between the diagnosis of the disease and the date on which the euthanasia took place, is not statistically significantly different between high and low-incidence regions. Most often it was between half a year and two years after the diagnosis (28–30%), other time spans were; between two and 10 years (24–26%), between a month and a half year (17–23%), more than 10 years (5–6%) and less than a month (1%).

#### Time between earliest euthanasia intention and death

We also looked at when a patient first expressed some form of a euthanasia intention, either in the form of a written directive or in the form of an oral mention. The mean/median time between that first expression and the euthanasia is longer in low-incidence regions (890/280 days) than in high-incidence regions (753/81 days). The categorical data show in more detail that a statistically higher percentage of individuals from high-incidence regions received euthanasia on a shorter notice after they had first mentioned their desire (19% receive euthanasia within 6 months after their first intention, versus 11% of the patients from low-incidence regions).

#### Time between actual euthanasia request and death

Apart from the aforementioned ‘first intention,’ we have looked at the time between a patient’s actual request (namely, to have a euthanasia wish being effectuated) and their death. This period was not significantly different between the high-incidence and low-incidence regions. Most often it was between 2 and 7 days (16%/18%, respectively). Other time spans were between one and two weeks (8%/12%), between a month and a half year (8%/9%), more than a year (2%) and one day (1%/2%).

#### First euthanasia intention: before or after a diagnosis?

Another question is the following order of, and the time span between diagnosis and first expression of the desire to receive euthanasia. No significant differences were found between the high and low-incidence regions. In both regions some individuals discussed euthanasia before they received a diagnosis. However, most of the time the first mention of euthanasia was well (i.e., on average half a year) after the diagnosis.

### The setting of euthanasia

#### Dying means

From our data it appears that a statistically significant higher percentage of individuals in high-incidence regions chose for euthanasia (physician administers lethal drugs), compared to low-incidence regions (*p* = 0,018). In low-incidence regions, it appears that more often the first choice was physician assisted suicide (the patient administers lethal drugs that were prescribed by the physician).

#### Place of death

In our dataset, and for practical reasons only, the place of death was listed for about 50% of the patients. Of these 50%, most received euthanasia at home (41.5% for high- and 35.3% for low-incidence regions). Other places of death: hospice (2.6/4.3%, nursing home (2.6/3.5%) and hospital (2/2.2%). None of these differences are statistically significant.

#### Physicians

As for the physicians, a distinction can be made between physicians who provided euthanasia or assisted suicide and consulting doctors.

*Specialty of the euthanizing physician.*

The vast majority of euthanizing physicians, both in high (93%) and in low (95%) incidence regions were general practitioners. There were no statistically significant regional differences in specialties.

*Specialty of the consulting physician*.

Even when it comes to the consulting physician, their most common specialty was general practitioner. However, in low-incidence regions, significantly more psychiatrists were involved as consulting doctors (*p* = 0,007). In cases in low-incidence regions, 78.4% of the consulting doctors were GPs, and 9.2% psychiatrists. In high-incidence regions these percentages were 82.1% GPs and 2.8% psychiatrists.

### Qualitative analysis of morally relevant themes

The frequency with which morally relevant themes occurred in the data gave us some indication of the most salient ethical questions in high- and low-incidence areas. The majority of ethically relevant aspects feature in largely the same frequency in both high- and low-incidence regions. For example, family involvement and pressure of some kind (which may involve relatives, patients themselves, and care professionals).

Only on two morally relevant aspects high and low-incidence regions seem to show differences. The first is mentions of a patient’s autonomy – often connected to their biography or previous profession – which may have played a decisive role for physicians to make conclusions about the unbearable character of the suffering and the seriousness of the request. Examples of such references are:

*“He has been in the military and has held positions in the business world with major responsibilities. His wife adds that he always had control and did not relinquish it,”* (consulting physician, euthanasia report)

*“He wants to keep everything under control. It’s never good enough. He once was a prisoner of war. That's where his urge for freedom and his tendency towards independence comes from,”* (consulting physician, euthanasia report)

*“He has been the director of a company and has always made decisions himself,”* (consulting physician, euthanasia report)

*“The patient has always been an independent, very erudite woman, has had to make many decisions in local politics and now wants to make the decision herself to say goodbye to life in a dignified manner.”* (consulting physician, euthanasia report)

We found that in low-incidence regions, such references to ‘autonomy’ in the dataset feature almost twice as frequent as in high-incidence regions.

Another difference is mentions of: ‘euthanasia performed by another doctor than a patient’s attending physician’. In low-incidence regions, the number of mentions is about twice as high as in high-incidence regions. As for the reasons for transferring the responsibility for performing euthanasia, the predominant motive in low-incidence regions was of a rather principal nature, reflected in comments such as:

*“I run a duo practice with my colleague who cannot perform euthanasia because of his religious beliefs. I am open to it, though.”* (attending physician, euthanasia report)

*“His GP has little experience with euthanasia and feels less confident to perform euthanasia in this situation, where there is not, for example, a terminal metastatic carcinoma.”* (attending physician, euthanasia report)

*“Her own attending physician does not want to be actively nor passively involved in euthanasia for reasons of principle.”* (attending physician, euthanasia report)

In high-incidence regions in the dataset, the reasons for involving a different doctor instead of the patient’s own fall almost exclusively under the header of ‘practical reasons,’ – e.g., because of conflicting calendars, or of illness or vacation on the part of the physician.

## Discussion

In this study aimed to compare euthanasia practices in high and low-incidence regions in the Netherlands. We looked specifically at differences in patient characteristics, the process, the setting, and some of the potentially moral relevancies. Some significant differences were found between high and low-incidence regions. Compared to low-incidence regions, high-incidence regions were characterized by patients being older at time of death, a shorter time span between patients’ first euthanasia request and their eventual death; patients’ more often having a GP as a consulting doctor, and euthanasia more frequently being the main dying means (as opposed to assisted suicide). The low incidence regions had somewhat younger patients, more patients with dementia, a longer life expectancy, more psychiatrists as consulting doctors, and more assisted suicides compared to the higher incidence regions.

### Interpretation

In the Netherlands, the reported euthanasia numbers have risen considerably in the past decades: from about 2,019 in 2005 [23] to 9,958 in 2024. Besides the rising numbers, the range of pathologies underlying an euthanasia request gradually expanded from terminal illnesses to chronic illnesses, including dementia, multiple geriatric syndromes and psychiatric disorders [15, 17, 21, 29], and there was a corresponding increase in the average life expectancy of patients requesting euthanasia. Also, the importance of individual autonomy has increased [24]. Previous studies on practice variation in euthanasia in the Netherlands showed that variation in euthanasia rates between regions decreased over time [10], suggesting low-incidence regions have a sharper increase of the euthanasia ratio than high-incidence regions.

On the basis of this knowledge – and in the virtual absence of both qualitative and quantitative research into regional variation about euthanasia –, we started out on the assumption that high-incidence regions may more reflect the newest developments, whereas the euthanasia practice in low-incidence regions would more reflect the situation in the earlier 2000s. After all, earlier research pointed out that low-incidence regions that ‘lag behind’ start to look like ‘early adopters’ over time [10]. More concretely, we expected differences in patient characteristics, the process, setting and morally relevant themes between high and low-incidence regions. Interestingly, we only found one of our expectations confirmed. We will discuss the details of the insights of this study accordingly.

#### Patient characteristics

Regarding *patient characteristics*, we expected that there would be a more diverse patient group (e.g. neuromuscular diseases, dementia, psychiatric diseases) in high-incidence regions than in low-incidence regions (mainly terminal, most often cancer patients). In the present study, however, we found that the patient groups in both regions were roughly the same, except that we found a higher percentage of dementia patients in low-incidence regions (nearly 6% vs. 2.5% of reported cases). This is particularly striking, given that 2.5% euthanasia with dementia is the national average [25]. Furthermore, we found no significant differences in gender (which is in line with previous research [5] and cause of suffering between the regions. As for their age, patients in high-incidence regions who received euthanasia were slightly older than those in low-incidence regions (70 versus 68).

#### Process

As for differences in *process*, we expected that euthanasia patients would have a longer average life expectancy in high-incidence regions. Contrary to what we expected, we found a higher average life expectancy in low-incidence regions (8 months versus almost 4 months). We believe that the (differences in) average life expectancy should be interpreted against the backdrop of the (differences in) diagnose types. For example, strikingly, there is a higher proportion of patients with dementia receiving euthanasia in low incidence regions, that also show a longer life expectancy at time of death. Typically, patients with dementia, who receive euthanasia, usually have a longer life expectancy [11].

#### Setting

When it comes to the *setting*, we found no significant differences in place of death and specialty of the euthanizing physician, but we did find a higher percentage of assisted suicides in low-incidence regions. For reasons that may have to be explored later, in low incidence regions patients and doctors choose more often for the method of self-administration.

As for the consulting physician, it appeared that in low-incidence regions psychiatrists were more frequently involved. As for the higher percentage of psychiatrists as consulting physicians, it is plausible that this may be related to the higher percentage of dementia cases (since the medical professional guideline strongly recommends consulting a second independent physician with expertise to determine capability of the patient, the diagnosis and alternative treatment strategies [22].

#### Morally relevant themes

Regarding the themes of potential moral relevancy, we also started out with some initial expectations. Firstly, we had expected that in high-incidence areas individual patient autonomy would play a more important role than in low-incidence regions. Secondly, that in low-incidence regions there will be fewer doctors willing to perform euthanasia, which would result in more euthanasia cases performed by other doctors than their own GP.

The first expectation was not affirmed. We found that the role of patient autonomy behind a euthanasia request was more predominant in the case descriptions from low-incidence regions than in their high-incidence counterparts. One possible explanation is that in a context with less doctors willing to provide euthanasia, and with a local ‘color’ of fewer euthanasia cases taking place, patients need a certain determined attitude in order to achieve their euthanasia wish granted, which explains the more frequent references to autonomy as one of the patients’ steering motives. This does not mean that patients in low-incidence regions die ‘more autonomously’ than in high-incidence regions. After all, the lower incidence of euthanasia in these regions may well imply local preferences for not having euthanasia. But *if* someone wants euthanasia, autonomy is more often invoked here than elsewhere.

As for the second expectation: we found more frequent mentions of other physicians than the patient’s attending physician performing euthanasia in low-incidence regions than in high-incidence regions. This expectation was the only one we found to be affirmed. In low-incidence regions, where fewer physicians are prepared to perform euthanasia, patients who insist on having this option are more likely to have to resort to another doctor.

### Strengths and limitations

The use of this unique dataset is both a strength and a weakness of this research. First we will discuss some complicating factors in using this dataset [1]. We will conclude with the unique aspects of this study and suggestions for future research.

First of all, because of the way in which the data set was created (in an iterative process), there may be unmeasured confounding. There almost certainly are variables that (partly) determine the differences found, but that we did not measure using this dataset. Most variables in the quantitative part of the dataset were steady, which means that they were unambiguous and did not change in interpretation throughout the process of data gathering. Only when it comes to the patient’s natural life expectancy, the data may be of limited accuracy. Not only is it notoriously difficult for physicians to estimate how much time a patient has left, but (and probably precisely for this previous reason) also the question about the patient’s life expectancy disappeared from the standard reporting form in about 2010. From then on, the life expectancy in the dataset was inferred from other elements in the medical record. For example: being bedridden in a patient with metastatic cancer, or patients receiving heavy pain killers, sedatives, or oxygen are indications of a short natural life expectancy. Secondly, errors in variable measurement might have occurred, mainly in the qualitative part investigating the morally relevant themes. The aspects that were selected were directly linked to the legal due care criteria. There may have been changes (over time) in the way the ethicist (TAB) coded different variables. There might have been a bias toward common ethical themes in the political debate on euthanasia. Through moral reflection over the years, emphasis may have shifted. There are shifting panels in euthanasia practice over the years, for example to be seen in a shift in interpretation of the due care criteria as suggested in the Fourth Legal Evaluation [24], according to which patient autonomy is increasingly central in the interpretation of the due care criteria. This may have played into which themes were interpreted as morally relevant during the data gathering process for more than a decade.

Thirdly, there are biases in the creation of study population. The dataset dates from the period 2008–2014 and only contains about 40% of the reported euthanasia cases in the region Midden, as the remaining 60% were assessed by deputy ethicist-members. Still, 40% provides a representative sample. Even with only data on euthanasia cases from region Midden (which contains about 1/5th of the Netherlands), it was possible to make a clear distinction by selecting high and low-incidence regions. Lastly, although information about patient, process, and setting were a standard part of the information that physicians were expected to report to the regional review committee, this does not apply to references to ‘morally relevant themes.’ Since in the standard reporting form there is no obligation for doctors to provide information, and since these ‘morally relevant aspects’ are reported by one committee member only (TAB) – albeit in and after dialoguing with the other committee members –, these remarks are of limited quantitative value. We chose to analyze the morally relevant themes qualitatively because these data are too sensitive to biases (errors in variable measurement) and moral themes are too nuanced to categorize or quantify.

Finally, because the dataset grew over a long period of time (2008–2014), and also for reasons of privacy, no contextual factors were measured or denoted, that could have shed light on the regional differences observed (e.g. data on health care accessibility). On the other hand, where this has been done in other studies [10], the uniqueness of our dataset is on different content that stems directly from euthanasia files.

Acknowledging complicating factors, we still used this data because it is unique in its sort. The use of individual data allowed us to look at regional variation in characteristics of performed euthanasia cases (patient characteristics, timelines, setting and morally relevant themes). Neither the regional review committees nor the five-yearly governmental evaluations provide information of the kind that we have analyzed here. Earlier intentions in the five-yearly evaluations to include such in-depth research have not materialized, in part because of the enormous effort involved. For the first time the black box of local practices in the Dutch euthanasia practice was opened, which leads us to new insights and new questions.

### Future research

Research on practice variation in end-of-life care and euthanasia/PAS should be more focused in opening the black box of local practices: what practices underly or characterize the regional differences? In addition to quantitatively examining and interpreting practices, qualitative research is needed in which personal considerations in individual cases are discussed with doctors and patients. For example, through interviews and focus groups with physicians in high and low incidence regions we can shed light on local practices. In what ways is the topic of euthanasia discussed in the doctor’s office? What are care trajectories leading to eventually choosing euthanasia? Why do people opt for euthanasia or rather consider physician assisted suicide? What are characteristics of other end of life care decisions? What about the accessibility of alternatives, such as palliative care or hospice care? The existing body of knowledge from sociological and anthropological research should be used in in depth (qualitative) research. Only by getting more insight into everyday practices in regions, can we promote appropriate and high-quality care at the end of life in general and euthanasia/PAS in specific.

## Data Availability

Data are available upon reasonable request.

## Appendix 1

**Table:**

High-incidence municipalities	Euthanasia rate	Records in dataset	Low-incidence municipalities	Euthanasia rate	Records in dataset
Bunnik	7,5%	9	Bunschoten	0,5%	1
Heumen	7,1%	18	Staphorst	0,6%	0
Almere	7%	134	Zwartewaterland	0,6%	1
Beuningen	6%	25	Oldebroek	1%	2
Borne	5,7%	27	Kampen	1%	4
Druten	5,7%	13	Vijfheerenlanden	1%	15
Soest	5,6%	54	Dinkelland	1,2%	10
Stichtse Vecht	5,1%	59	Twenterand	1,2%	7
Nijmegen	5%	154	Elburg	1,3%	3
Losser	4,7%	17	Hardenberg	1,3%	15
**Total**		**510**	**Total**		**58**
Renkum	4,6%	34	Rijssen Holten	1,5%	4
Houten	4,5%	18	Raalte	1,5%	8
Nieuwegein	4,5%	44	Ermelo	1,5%	16
Duiven	4,5%	12	Noordoostpolder	1,6%	14
Wijk bij Duurstede	4,4%	17	Nunspeet	1,6%	9
De Bilt	4,4%	36	Putten	1,6%	0
Baarn	4,4%	19	Veenendaal	1,7%	12
Hof van Twente	4,3%	32	Barneveld	1,7%	13
Hengelo	4,3%	78	Neder Betuwe	1,8%	3
De Ronde Venen	4,3%	30	Wageningen	1,8%	16
**Total**		**320**	**Total**		**95**
**Total**		**830**	**Total**		**153**

Percentages from appendix 1 are based on the database used in the research of Groenewoud and colleagues (2021). The euthanasia rate is calculated by the ratio between the number of deaths over the period 2013-2017 and the number of euthanasia cases over the period 2013-2017. The dataset can be retrieved upon reasonable request at the first author (SG)
